# Skeletal muscle predicts ventilator-free days, ICU-free days, and mortality in elderly ICU patients

**DOI:** 10.1186/cc12901

**Published:** 2013-09-19

**Authors:** Lesley L Moisey, Marina Mourtzakis, Bryan A Cotton, Tahira Premji, Daren K Heyland, Charles E Wade, Eileen Bulger, Rosemary A Kozar

**Affiliations:** 1 Department of Kinesiology, University of Waterloo, Waterloo, ON, Canada; 2 Department of Surgery, University of Texas Health Science Center, Houston, TX, USA; 3 Center for Translational Injury Research, University of Texas Health Science Center, Houston, TX, USA; 4 Department of Medicine, Queen’s University, Kingston, ON, Canada; 5 Department of Surgery, Harborview Medical Center, University of Washington, Seattle, WA, USA

## Abstract

**Introduction:**

As the population ages, the number of injured elderly is increasing. We sought to determine if low skeletal muscle mass adversely affected outcome in elderly patients following trauma.

**Methods:**

Patients ≥ 65 years of age with an admission abdominal computed tomography scan and requiring intensive care unit (ICU) stay at a Level I trauma center in 2009–2010 were reviewed. Muscle cross-sectional area at the 3rd lumbar vertebra was quantified and muscle index, a normalized measure of muscle mass, was calculated and related to clinical parameters including ventilator-free days, ICU-free days, and mortality. Using previously established sex-specific, muscle index cut-points, patients were then categorized as sarcopenic or non-sarcopenic and differences in clinical outcomes between these two groups were also compared. We also examined muscle index as a continuous variable relative to the same clinical outcomes.

**Results:**

There were 149 severely injured elderly patients (median age 79 years) enrolled in this study of which 71% were sarcopenic. Of the patients who were sarcopenic, 9% were underweight, 44% normal weight, and 47% overweight/obese as per body mass index (BMI) classifications. The overall mortality rate was 27% and univariate analysis demonstrated higher mortality among those who were sarcopenic (32% vs. 14%, *P* = 0.018). After controlling for age, sex, and injury severity, multiple logistic regression demonstrated that increased muscle index was significantly associated with decreased mortality (OR per unit muscle index = 0.93, 95% CI: 0.875-0.997, *P =* 0.025). In addition, multivariate linear regression showed that sarcopenia, but not muscle index, was associated with decreased ventilator-free (*P* = 0.004) and ICU-free days (*P* = 0.002). Neither BMI, serum albumin nor total adipose tissue on admission were indicative of survival, ventilator-free or ICU-free days.

**Conclusions:**

Sarcopenia is highly prevalent in the elderly population with traumatic injuries. Traditional measures of nutritional assessment, such as BMI and serum albumin, do not accurately predict outcome in the injured elderly. Sarcopenia, however, represents a potential new predictor for mortality, discharge disposition, and ICU utilization. Measurement of muscularity allows for the early identification of at-risk patients who may benefit from aggressive and multidisciplinary nutritional and rehabilitative strategies.

## Introduction

Elderly patients admitted to the ICU experience worse clinical outcomes, including higher mortality rates and longer hospital length of stay (LOS) than nonelderly patients [1-4]. Although the injured elderly also experience a higher mortality rate than younger patients [5], the majority of injured elderly admitted to the ICU survive to discharge [6]. Of the elderly patients who survive in the ICU as well as hospitalization, most demonstrate impaired physical, cognitive and social functioning [4,7,8], which may continue during recovery and pose a drain on healthcare resources [9]. The presence of other comorbidities, poor nutritional status and other factors may exacerbate clinical and functional outcomes in survivors.

Traditionally, malnutrition at hospital admission has been determined by multiple factors that may include body mass index (BMI) and serum albumin. Albumin is typically a poor marker of nutritional status [10], and, in the acute trauma setting, albumin may be affected by changes in intravascular volume and other factors, thus making it a less reliable predictor of nutritional status. Low BMI (<18.5 kg/m^2^) is also strongly related to increased mortality in elderly patients who have been admitted to the hospital [11]. However, BMI is a crude measurement based on height and weight and provides an assessment of body size without any distinction between different tissues (for example, lean versus adipose tissue). Poor nutrition during hospital stay in the critically ill [12] (which includes elderly trauma patients; see [13]), as well as prolonged immobility as a consequence of critical illness, may lead to greater risk for muscle loss during the ICU stay [14]. Skeletal muscle mass is highly important in immune function, glucose disposal, protein synthesis and mobility; therefore, decreases in skeletal muscle can result in a plethora of physiologic impairments [15-17]. Fat mass may also be important to clinical outcomes. In the elderly, increased fat mass has been associated with decreased mortality [18], whereas in a population comprising younger and older adults, increased fat mass was associated with increased LOS [19].

We therefore sought to determine whether low muscularity and low adiposity adversely affect outcomes in elderly patients admitted to the ICU following trauma. We hypothesized that low muscle mass would be prevalent in injured elderly patients at the time of ICU admission and that those patients with low muscle mass and/or low fat mass would have poorer clinical outcomes.

## Materials and methods

### Patient selection

This study was approved by the Institutional Review Board (IRB) of the University of Texas Health Science Center at Houston and the University of Waterloo Research Ethics Board. The study was determined to qualify for exempt status by the IRB because data were collected from existing patient records. Therefore, the need for patient consent to participate was waived.

Injured elderly patients (≥ 65 years) admitted to the ICU at Texas Memorial Hermann Hospital (level 1 trauma center) in 2009 and 2010 were identified. Of the identified patients, those for whom we had a computed tomography (CT) scan of the abdomen for diagnostic purposes on the day of admission were included, and their deidentified CT images were analyzed for skeletal muscle and adipose tissue cross-sectional areas.

### Physical characteristics and variables

Demographic data parameters obtained included age; sex; mechanism of injury; head, chest and abdomen abbreviated Acute Injury Scale (AIS) score; Injury Severity Scale (ISS) score; admission serum albumin level; admission systolic blood pressure (SBP); body temperature; base deficit; hemoglobin and hematocrit levels; international normalized ratio (INR); height (in centimeters); weight (in kilograms); length of hospital stay and ICU stay; number of days requiring mechanical ventilation; and mortality. Height and weight were used to calculate BMI (calculated as weight in kilograms divided by height in meters squared), allowing for classification of weight categories as follows: underweight (BMI <18.5), normal (BMI 18.5 to 24.9), overweight (BMI 25 to 29.9), and obese (BMI >30 kg/m^2^) [20].

### Computed tomography image analysis

Skeletal muscle and adipose tissue cross-sectional areas were quantified using single-slice CT scans at the third lumbar vertebra (L3). Tissue cross-sectional areas at this landmark are strongly correlated to whole-body muscle and adipose tissue mass distribution [21-23]. Skeletal muscle tissues quantified included the psoas, erector spinae, quadratus lumborum, transverse abdominal, internal and external obliques and rectus abdominus. Adipose tissue compartments analyzed included subcutaneous (SAT), visceral (VAT) and intramuscular (IMAT). Summation of these compartments allowed for quantification of total adipose tissue (TAT).

CT scans were analyzed for body composition using sliceOmatic version 4.3 image analysis software (TomoVision, Montreal, QC, Canada). This software allows the precise quantification of specific tissues using Hounsfield unit thresholds defined at −29 to 150 for skeletal muscle [24], −150 to 50 for VAT [25] and −190 to −30 for both SAT and IMAT [24]. Cross-sectional area in centimeters squared was computed for each tissue by summing tissue pixels and multiplying by pixel surface area. All CT images were analyzed by a trained analyst. Intra- and inter-analyst coefficients of variation were calculated. For the intra-analyst coefficient of variation, the primary analyst reanalyzed 10 random scans approximately 7 months after the time that the original scans were analyzed. A second analyst analyzed 90 randomly selected CT scans, and inter-analyst coefficients of variation were calculated and gave the following results: 1.2%, 3.4%, 0.95%, 0.68% and 0.51% for skeletal muscle, IMAT, VAT, SAT and TAT, respectively. The mean intra-analyst coefficients of variation for skeletal muscle, IMAT, VAT, SAT and TAT were 0.37%, 2.0%, 0.67%, 0.48% and 0.49%, respectively. These inter- and intra-analyst coefficients of variation are in the same ranges described in previous studies in which these specific tissues have been examined [26,27].

To normalize the diversity in patients’ height, skeletal muscle cross-sectional area at L3 (in centimeters squared) was divided by height in meters squared. This value is denoted as muscle index (centimeters squared divided by meters squared), with a lower value representing lower muscle mass. Using the muscle index, patients were also categorized as sarcopenic or nonsarcopenic based on previously reported muscle index cut-points, less than 38.9 cm^2^/m^2^ for females and less than 55.4 cm^2^/m^2^ for males [21]. Those below these cut-points were identified as sarcopenic.

Similarly to those used to quantify skeletal muscle, regression equations have been derived from a healthy population to estimate VAT [23] and from an advanced cancer population to estimate TAT [21] from single-slice cross-sectional areas. VAT and TAT cross-sectional area indices were normalized based on patient height. Cut-points for identifying patients with low VAT and/or low TAT do not currently exist. For the purposes of this study, the median VAT and TAT index values were determined for males and females and used as arbitrary cut-points to distinguish high- and low-fat masses. VAT and TAT indices were also examined as continuous variables.

### Statistics

Continuous data are presented as medians with 25th and 75th interquartile ranges (IQRs), with comparisons between the groups performed using the Wilcoxon rank-sum test (Mann–Whitney *U* test). Categorical data are reported as proportions and, where appropriate, tested for significance using a χ^2^ test or Fisher’s exact test. All statistical tests were two-tailed, and *P* ≤ 0.05 was set as the statistical significance level.

Purposeful regression modeling was then used to construct a multivariate linear regression model and then a multivariate logistic regression model to evaluate outcomes in patients according to different measures of nutritional status [28]. In an effort to minimize the risk of falsely identifying significant results with multiple comparisons, all variables were specified and judged *a priori* to be clinically sound. These independent variables included age, sex, ISS score, arrival vital signs, and laboratory values. After these variables were calculated, they were entered into stepwise regression that generated three variables of significance (age, sex and ISS score). These were then applied in a sequential (rather than simultaneous) fashion to both multivariate logistic and linear regression analyses to evaluate these variables and admission values of nutritional status. STATA version 10.0 software (Stata Corp, College Station, TX, USA) was used for statistical analysis.

## Results

### Physical characteristics

One hundred fifty-three elderly patients who were admitted to the ICU following injury had an abdominal CT scan that encompassed L3 and could subsequently be used for evaluation of muscle and fat depots. TAT could not be calculated for four patients, as SAT was outside the CT viewing field and thus could not be quantified, so these patients were excluded from further analysis. Of the remaining 149 patients (85 males and 64 females), overall, patients had a median BMI of 25.6 kg/m^2^ (IQR = 22.7 to 28.2) (Table 1). On the basis of BMI, 57% of all patients were overweight and/or obese, whereas only 7% were underweight. In contrast, CT image analysis demonstrated that 106 (71%) of 149 patients were sarcopenic at the time of ICU admission, independent of their BMI. In the sarcopenic group, 38% were characterized as overweight and 9% were characterized as sarcopenic and obese (patients who were obese concurrently categorized as sarcopenic). Sarcopenic patients had significantly lower total fat mass compared with nonsarcopenic patients (*P* = 0.016) (Table 1).

**Table 1:** Physical characteristics of injured elderly patients at ICU admission^a^

**Characteristics**	**All patients**	**Sarcopenic**	**Nonsarcopenic**	***P*-value**
	**(*N *** **= 149)**	**(*n *** **= 106)**	**(*n *** **= 43)**	
BMI (kg/m^2^)	25.6 (22.7 to 28.2)	24.4 (21.7 to 27.3)	27.6 (25.5 to 30.4)	<0.001
Underweight (%)	7 (n = 10)	9 (n = 9)	2 (n = 1)	0.13
Normal weight (%)	37 (n = 55)	44 (n = 47)	19 (n = 8)	0.004
Overweight (%)	42 (n = 62)	38 (n = 40)	51 (n = 22)	0.14
Obese (%)	15 (n = 22)	9 (n = 10)	28 (n = 12)	0.003
Muscle cross-sectional area (cm^2^/m^2^)				
All patients	42.7 (36.1 to 49.3)	38.8 (34.0 to 45.7)	50.7 (43.3 to 56.9)	<0.001
Estimated whole-body muscle mass (kg)	23.8 (19.5 to 27.6)	23.1 (18.9 to 26.8)	25.6 (21.7 to 32.9)	0.002
Total fat cross-sectional area (cm^2^/m^2^)	108 (73.4 to 146)	98.8 (70.3 to 135)	131 (95.8 to 177)	<0.001
Estimated whole-body total fat mass (kg)	24.3 (19.6 to 29.9)	22.8 (19.4 to 28.9)	26.3 (22.3 to 34.1)	0.016
Visceral fat cross-sectional area (cm^2^/m^2^)	43.5 (25.4 to 70.6)	41.7 (21.3 to 71.0)	52.0 (33.8 to 70.6)	0.24
Estimated visceral fat mass (kg)	2.8 (1.5 to 4.5)	2.8 (1.3 to 4.5)	2.7 (2.0 to 4.1)	0.63

^a^BMI = body mass index. Data for BMI, muscle cross-sectional area, estimated whole-body muscle mass, total fat cross-sectional area, estimated total fat mass, visceral fat cross-sectional area and estimated visceral fat mass are presented as medians (interquartile ranges). *P*-values depict the statistical difference between sarcopenic and nonsarcopenic patients, with statistical significance set at *P* ≤ 0.05.

Patients in the whole sample were severely injured with an average ISS score of 19 (Table 2). The overall median age was 79 years (IQR = 72 to 85), with sarcopenic patients being significantly older (*P* = 0.007). The most common mechanism of injury was motor vehicle collision, followed by same-level falls (Table 2). There were no differences between sarcopenic and non-sarcopenic patients in terms of the following parameters: ISS score; AIS score by body region for head, chest and abdomen; admission blood pressure; base deficit; albumin; and coagulation. However, sarcopenic patients tended to have a higher incidence of falls from standing position (*P* = 0.055) (Table 2). Nonsarcopenic patients had significantly lower admission hemoglobin (*P* = 0.016) (Table 2).

**Table 2:** **Diagnostic characteristics of injured elderly patients at ICU admission**^**a**^

**Characteristics**	**All patients**	**Sarcopenic**	**Nonsarcopenic**	***P*-value**
	**(*N *** **= 149)**	**(*n *** **= 106)**	**(*n *** **= 43)**	
Age (yr)	79 (72 to 85)	80 (73 to 86)	76 (68 to 83)	0.007
Sex				
Male (%)	57 (*n* = 85)	67 (*n* = 71)	33 (*n* = 14)	<0.001
Female (%)	43 (*n* = 64)	33 (*n* = 35)	67 (*n* = 29)	
Mechanism of injury				
MVC (%)	51 (*n* = 76)	47 (*n* = 50)	60 (*n* = 26)	0.150
Fall (%)	37 (*n* = 60)	45 (*n* = 48)	28 (*n* = 12)	0.055
Automobile–pedestrian (%)	4 (*n* = 6)	5 (*n* = 3)	10 (*n* = 4)	0.26
Other (%)	8 (*n* = 12)	3 (*n* = 5)	2 (*n* = 1)	0.73
ISS score	19 (14 to 26)	20 (14 to 29)	17 (13 to 24)	0.17
Low-energy mechanism (%)	45 (*n* = 67)	50 (*n* = 53)	30 (*n* = 13)	0.026
Head AIS score	3 (3 to 4)	4 (3 to 4)	3 (3 to 4)	0.083
Chest AIS score	3 (3 to 4)	3 (3 to 3)	3 (3 to 3)	0.14
Abdomen AIS score	2 (2 to 3)	2 (2 to 3)	3 (2 to 3)	0.51
ED SBP (mmHg)	137 (110 to 161)	136 (108 to 158)	142 (124 to 161)	0.21
ED temperature (°F)	97.2 (96.4 to 98.1)	97.2 (96.3 to 98.1)	97.3 (96.9 to 98.2)	0.24
ED base value	−1.5 (−5 to 2)	−1.5 (−4 to 2)	−1.5 (−6.5 to 2.5)	0.94
ED hemoglobin (g/dl)	9.7 (7.6 to 12.6)	11.8 (7.7 to 13.1)	7.8 (7.4 to 9.7)	0.016
ED INR	1.28 (1.09 to 1.55)	1.24 (1.08 to 1.52)	1.43 (1.16 to 2.08)	0.18
ED albumin (g/dl)	3.4 (3.2 to 3.8)	3.4 (3.1 to 3.8)	3.5 (3.2 to 3.6)	0.63

^a^AIS = Acute Injury Scale score, ED = Emergency Department, F = Fahrenheit, INR = international normalized ratio, ISS = Injury Severity Scale score, MVC = motor vehicle collision, SBP = systolic blood pressure. Data are presented as medians (interquartile ranges). The following data are presented as medians (interquartile ranges): age, ISS score, head AIS score, chest AIS score, abdomen AIS score, ED SBP, ED body temperature, ED base value, ED hemoglobin, ED INR and ED albumin. *P*-values depict statistical differences between sarcopenic and nonsarcopenic patients, with statistical significance set at *P* ≤ 0.05.

### Low skeletal muscle mass relates to poor clinical outcomes

The overall mortality rate in the study population was 27%. More than twice the number of sarcopenic patients died in the hospital compared with nonsarcopenic patients (32% versus 14%; *P* = 0.018) (Table 3). Of the nonsarcopenic patients who survived, 26% were discharged to home, compared with only 14% of sarcopenic patients (*P* = 0.085). After controlling for age, sex and ISS score, multivariate logistic regression analysis demonstrated that decreased muscle index, but neither BMI nor serum albumin, was significantly associated with in-hospital mortality (odds ratio (OR) = 0.93, 95% confidence interval (CI) = 0.875 to 0.997; *P =* 0.025) (Table 4). As such, every unit increase in muscle index is associated with a 7% OR reduction in mortality.

**Table 3:** Relationship of mortality to sarcopenia, total adiposity and visceral adiposity

**Group classification**	**Proportion of deceased patients**	***P*-value for proportion of deceased individuals between each group**^**a**^
Sarcopenic	32% (34/106)	0.018^b^
Nonsarcopenic	14% (6/43)	
Low total fat mass	32% (25/77)	0.11^c^
High total fat mass	21% (14/68)	
Low visceral fat mass	33% (26/78)	0.18^d^
High visceral fat mass	20% (14/71)	

^a^Statistical significance was accepted at *P* ≤ 0.05. ^b^*P*-value for proportion of deceased individuals between sarcopenic and nonsarcopenic patients. ^c^*P*-value for proportion of deceased individuals between patients with low and high total fat mass. ^d^*P*-value for proportion of deceased individuals between patients with low and high visceral fat mass.

**Table 4:** **Multiple logistic regression model predicting survival in hospital after controlling for age, sex and Injury Severity Scale score**^**a**^

**Measurement**	**Odds ratio**	**95% CI**	***P*-value**
Muscle index	0.93	0.875 to 0.997	0.025
TAT index	0.99	0.99 to 1.001	0.11
BMI (kg/m^2^)	0.97	0.86 to 1.09	0.62
Albumin (g/dl)	3.75	0.63 to 22.41	0.15

^a^BMI = body mass index, TAT = total adipose tissue. Statistical significance was accepted at *P* ≤ 0.05.

After controlling for age, sex and injury severity, multivariate linear regression analysis demonstrated that neither muscle index nor BMI was significantly associated with ventilator-free and ICU-free days (*P* = 0.19 and 0.36, respectively, for muscle index; *P* = 0.85 and 0.75, respectively, for BMI) (Table 5). In contrast, when we compared patients who were sarcopenic with those who were nonsarcopenic, we found that sarcopenic patients had significantly lower ventilator-free days (median = 19 (IQR = 0 to 28) vs. median = 27 (IQR = 18 to 28), respectively; *P* = 0.004) and significantly lower ICU-free days (median = 19 (IQR = 0 to 25) vs. median = 16 (IQR = 0 to 24), respectively; *P* = 0.002).

**Table 5:** **Multivariate linear regression model predicting ventilator-free and ICU-free days after controlling for age, sex and Injury Severity Scale score**^**a**^

**Measurement**	**Coefficient of correlation**	**95% CI**	***P*-value**
Ventilator-free days			
Muscle index	0.060	−0.025 to 0.126	0.19
TAT index	−0.004	−0.026 to 0.017	0.71
Albumin	5.49	−0.648 to 11.618	0.078
BMI	−0.043	−0.49 to 0.40	0.85
ICU-free days			
Muscle index	0.032	−0.037 to 0.101	0.36
TAT index	−0.002	−0.022 to 0.017	0.82
Albumin	5.32	0.032 to 10.61	0.049
BMI	−0.065	−0.47 to 0.34	0.75

^a^BMI = body mass index, CI = confidence interval, TAT = total adipose tissue, VAT = visceral adipose tissue. Statistical significance was accepted at *P* ≤ 0.05.

### Lower fat mass is not correlated with poor clinical outcomes

Overall, patients had a median total fat mass of 24.3 kg (IQR 19.6 to 29.9). Patients with sarcopenia had significantly less total fat than those who were not sarcopenic (22.8 kg vs. 26.3 kg; *P* = 0.016) (Table 1). There were no differences in VAT mass between sarcopenic and nonsarcopenic patients (*P = *0.63) (Table 1). The median VAT index (60.8 and 32.5 cm^2^/m^2^ for males and females, respectively) and TAT index (115.6 cm^2^/m^2^ for males and 95.5 cm^2^/m^2^ for females) were used as arbitrary cut-points to distinguish high and low fat masses. After controlling for age, sex and injury severity, multiple logistic regression demonstrated that a low TAT index was not associated with mortality (*P* = 0.11) (Table 4).

Patients with low versus high TAT had a median of 17 ventilator-free days (IQR = 0 to 28) and 25 ventilator-free days (IQR = 0 to 28), respectively (*P* = 0.54), and 11 ICU-free days (IQR = 0 to 24) and 20 ICU-free days (IQR = 0 to 25), respectively (*P* = 0.31). Patients with low VAT had 26 ventilator-free days (IQR = 2 to 28) versus 25 ventilator-free days (IQR = 0 to 28) for those with high VAT (*P* = 0.55). Similarly, median ICU-free days were 23 (IQR = 0 to 25) and 19 (IQR = 0 to 25) for patients with low VAT and high VAT, respectively (*P* = 0.69). After controlling for age, sex and injury severity, multivariate linear regression analysis demonstrated that TAT index was not significantly associated with ventilator-free and ICU-free days (*P* = 0.71 and *P* = 0.82, respectively) (Table 5).

## Discussion

We conducted a study of severely injured elderly patients admitted to the ICU and found that, at the time of admission, 71% were sarcopenic based on sex-specific cut-points [21]. Importantly, patients identified as sarcopenic had significantly increased mortality and decreased ventilator-free and ICU-free days. Interestingly, despite the frequency of sarcopenia in our injured elderly population, 7% of the patients were underweight, 37% were normal weight and 57% were overweight or obese as defined by BMI. Neither BMI nor serum albumin upon admission were predictive of survival, ventilator-free days or ICU-free days.

CT scans are frequently performed in trauma patients [29]. Single-slice CT images in the L3 region can predict whole-body muscle and adipose tissue volume in healthy [22,23] and diseased [21,30] populations. To the best of our knowledge, this study is the first in which CT imaging has been used for precise body composition analysis of critically injured patients. Other clinical populations, such as cancer cohorts, who tend to experience disease-related muscle atrophy, have exhibited a prevalence of sarcopenia of about 50% [21,30,31], less than the incidence in the injured elderly patients in our study. CT images are a powerful tool with which to advance our understanding of muscle and adipose tissue losses and their implications for clinical outcomes.

Traditional measures of weight, BMI and serum albumin are typically used to assess nutritional status and nutrient requirements of patients at admission and to detect changes during ICU and hospital stays. The present study suggests that at-risk patients may be overlooked using these traditional indicators or nutritional status. Muscularity therefore represents a potential new marker for identifying mortality risk but, more importantly, permits the early identification of patients who may benefit from aggressive and integrated nutrition and rehabilitative therapies.

After trauma in particular, muscle atrophy may be further exacerbated with prolonged bed rest, iatrogenic malnutrition, immobilization of fractured extremities, multiple surgeries as a result of injury and hypermetabolism, all of which are factors associated with sarcopenia. Acquired weakness following any prolonged critical illness impairs functional capacity and is an important contributor to the ongoing morbidity experienced by survivors of critical illness [9]. Skeletal muscle is also highly important in regulating immune function, glucose disposal and protein synthesis. Loss of lean tissue is specifically correlated with morbidity, increased hospital LOS and mortality [16,32,33]. Though the injured elderly admitted to the ICU receive inadequate nutrition [13], we do not know the extent to which optimizing nutrition after injury will have on muscle mass, as malnutrition is only one factor contributing to sarcopenia [34]. Skeletal muscle atrophy can impair cytokine [15] and insulin signaling, which may result in glucose intolerance [35,36] because more than 75% of glucose is handled by skeletal muscle [37]. Sarcopenia itself is associated with an increased risk of developing nosocomial infections [38]; however, we did not analyze nosocomial infections in this study. Sarcopenic patients had a higher incidence of falls from standing position. It is possible that decreased muscle mass may have contributed to postural instability, thus contributing to these findings.

Though increased fat mass is typically associated with various comorbidities, including diabetes and cardiovascular disease [39], increased fat mass appears to be protective and is associated with decreased risk of adverse events in hospitalized ICU patients [18]. In the present study, there were no overall associations between total and visceral fat mass with ventilator-free and ICU-free days.

### Methodological limitations

Despite the novelty of the use of CT images to identify specific features of body composition, future work is warranted to prospectively evaluate body composition using this methodology, accompanied by metabolic measures, to better understand the clinical implications of low muscle and fat mass. In addition, the prevalence of overweight and/or obese sarcopenic patients is of particular interest, given the increasing rate of obesity. Accurate documentation of comorbidities is important for future study designs, as comorbidities could contribute to poor clinical outcomes in these patients and were not controlled for in our present analyses.

Although sarcopenia has been reported in the elderly, this study was the first, to the best of our knowledge, to examine the implications of low muscularity or sarcopenia in injured, elderly ICU patients. CT is not performed on every ICU patient, as it is expensive and involves considerable radiation exposure for prospective evaluation of body composition [40]. However, the use of CT in the initial evaluation of severely injured patients is common, opening the possibility of early assessment of muscularity in this patient population. Preliminary data published by Gruther *et al*. [32] suggest that muscle thickness may negatively correlate with ICU LOS, but no attempts have been made to correlate muscle thickness on ultrasound with that measured by CT. This area is one that we are currently investigating in a prospective, multicenter study.

## Conclusions

We used the precision of CT to quantify muscle and fat mass in a cohort of injured, elderly ICU patients. We found that 71% of these patients were sarcopenic at admission, despite the fact that BMI identified only 9% of patients as underweight. Interestingly, 47% of sarcopenic patients were overweight or obese based on BMI measurements, which is a large proportion of patients who may be at risk for malnutrition. BMI is a parameter that is commonly used to screen nutritional status and calculate nutrient requirements. However, it fails to account for this large cohort of patients with low muscularity who may benefit from more aggressive and integrated nutritional and rehabilitative strategies to attenuate in-ICU muscle loss. The presence of sarcopenia had negative implications on mortality, ventilator-free days and ICU-free days. The use of CT to identify patients who have low muscularity is an important tool for future investigations that aim to improve clinical outcomes in injured, elderly ICU patients.

### Key messages

• Low muscle mass and/or sarcopenia are very prevalent in geriatric trauma patients and are associated with increased mortality.

• Sarcopenia is associated with decreased ventilator-free days and ICU-free days.

• Traditional measures of nutritional assessment (that is, BMI, serum albumin) do not accurately predict outcomes in elderly patients admitted to the ICU.

• Diagnostic CT scans are a useful tool for identifying specific features of body composition and relating them to clinical outcomes.

## Abbreviations

AIS: Acute injury scale; BMI: Body mass index; CT: Computed tomography; ED: Emergency department; F: Fahrenheit; IMAT: Intramuscular adipose tissue; INR: International normalized ratio; IQR: Interquartile range; ISS: Injury severity score; L3: Third lumbar vertebra; LOS: Length of stay; MVA: Motor vehicle accident; OR: Odds ratio; SAT: Subcutaneous adipose tissue; SBP: Systolic blood pressure; TAT: Total adipose tissue; VAT: Visceral adipose tissue.

## Competing interests

The authors declare that they have no competing interests.

## Authors’ contributions

LM participated in data analysis and interpretation as well as manuscript preparation. MM participated in the design of the study, data analysis and interpretation and manuscript preparation. BC performed the statistical analysis and participated in manuscript preparation. TP participated in data analysis and interpretation and manuscript preparation. CW, EB and DH participated in the design of the study and manuscript preparation. RK participated in the study design and was involved in data collection, analysis and interpretation and helped to prepare the manuscript. All authors read and approved the final manuscript.
